# Molecular diagnostic markers of *Tachysurus fulvidraco* and *Leiocassis longirostris* and their hybrids

**DOI:** 10.1186/s40064-016-3766-0

**Published:** 2016-12-30

**Authors:** Hongwei Liang, Shanshan Guo, Xiangzhong Luo, Zhong Li, Guiwei Zou

**Affiliations:** 1 Yangtze River Fisheries Research Institute, The Chinese Academy of Fisheries Sciences, No. 8, 1st Wudayuan Road, Wuhan East-lake Hi-tech Development Zone, Wuhan, 430223 China; 2 Freshwater Aquaculture Collaborative Innovation Center of Hubei Province, Wuhan, 430070 China

**Keywords:** Molecular diagnostic markers, COI, ITS, *Tachysurus fulvidraco*, *Leiocassis longirostris*

## Abstract

**Background:**

Bagridae is an important family of catfishes and has a high market demand. Recently, more cultivable Bagridae fishes are being exploited in China, and hybridization of some species has been carried out to achieve better growth performance, favorable sex ratios and better disease resistance. Yet, these hybrids have further increased the difficulties of taxonomy identification due to morphological indistinguishableness.

**Results:**

In this study, the molecular identification technologies for *Tachysurus fulvidraco*, *Leiocassis longirostris* sand their hybrids were successfully established by using mitochondrial *COI* and nuclear ITS sequences to identify the maternal and paternal lineage, respectively.

**Conclusion:**

These molecular diagnostic methods could also be used to manage breeding plans of hybrids, monitor and minimize the negative impacts of hybridization programs in aquaculture. Furthermore, our study could also provide a reference for establishing detection technique for hybrids in other groups of fishes.

## Background

 Bagridae is an important family of catfishes which belongs to Order Siluriformes and consists of more than 220 species of 21 genera (Ferraris 2007). It is commonly found in Africa and Asia and has abundant species diversity. In China, the family includes approximately 30 species of 4 genera which inhabit in Yangtze River, Pear River, Heilongjiang River and Yellow River (Qin et al. 2010). In the past decades, their populations have decreased rapidly and almost disappeared in many river systems due to over fishing, environmental pollution and other human disturbances (Luo et al. 2000; Wang et al. 2006; Xiao et al. 2014). In the meantime, an increasing number of Bagridae fishes have been cultured due to a high market demand. And to improve the growth performance and survival rate, some crossbreeding programs have been conducted, such as *Pseudobagrus ussuriensis* × *Pelteobagrus vachelli* (Cai et al. 2011; Qin et al. 2012; Wang et al. 2013), *Tachysurus fulvidraco* × *Pelteobagrus vachelli* (Wang et al. 2012; Zhang et al. 2016), *Tachysurus fulvidraco* × *Leiocassis crassilabrus*, and *Pelteobagrus vachelli* × *Leiocassis crassilabrus* (Wang 2013). The taxonomy of Bagridae is confusing and the validity of some catfishes should be further investigated and identified, what is more, taxonomic identification of hybrids is even more difficult. The main intention of crossbreeding is to obtain commercial advantages. Both yellow catfish (*Tachysurus fulvidraco*) and Chinese longsnout catfish (*Leiocassis longirostris*) in Bagridae have become important economic freshwater fishes with great market demand and high price in China due to their tender flesh, rich nutrition, few bones, convenient cooking and good taste (Liang et al. 2012; Xiao et al. 2014; Shen et al. 2014). To further improve the growth traits, they have also been used to produce hybrids.

Although crossbreeding could improve the genetic traits of animals, releases and escapes of hybrid individuals from fish farms have potential environment impacts. Hybrids can lead to the habitat changes for some fish species and change species composition in wild populations due to the change of diet or survival competition. Moreover, if the hybrids are fertile, the harmful effects of hybridization may cause pollution of gene pool and the extinction of some populations and species (Alllendorf et al. 2001). Therefore, accurate identification of fish hybrids is critical to the sustainable aquaculture development. Reliable identification methods of hybrids can not only serve for the effective management of crossbreeding practices but also are used to monitor their negative impacts. Hybrid authentication is usually difficult and uncertain by morphological methods. However, molecular identification method is an ideal and alternative method to the traditional morphological discrimination in hybrid identification (Scribner et al. 2000). In the present study, we will try to establish an effective identification method of *T. fulvidraco*, *L. longirostris* and their hybrids using the polymerase chain reaction (PCR) based on mitochondrial and nuclear molecular marker.

## Methods

### Experimental samples

In this study, 30 *T. fulvidraco* individuals and 30 *L. longirostris* individuals were used to analyze parental lineages. For the interspecific hybrid, 30 specimens of F_TL_ (crosses using *T. fulvidraco* as female and *L. longirostris* as male) and F_LT_ (crosses using *L. longirostris* as female and *T. fulvidraco* as male) were genetically analyzed. All samples were obtained from the experimental farm of Yangtze River Fisheries Research Institute, Chinese Academy of Fishery Sciences (YFI). The fish specimens were stored in the fish museum of YFI. All experimental procedures for the target fish were carried out in accordance to the standards of the Animal Care Policy of YFI.

### DNA extraction, PCR amplification and sequencing

Total genomic DNA was extracted from muscle tissue using the traditional phenol–chloroform extraction method (Taggart et al. 1992). DNA quality was determined by electrophoresis in a 1% agarose gel. Cytochrome C Oxidase subunit I (*COI*) sequences were obtained by the described primer pairs in Table 1 which were designed from the *COI* sequences of *T. fulvidraco* (GenBank accession no. HM641815) and *L. longirostris* (GenBank accession no. NC014586). For identifying the hybrid, the nuclear ITS sequences of *T. fulvidraco* (10 individuals) and *L. longirostris* (10 individuals) were firstly obtained by the published primer pairs ITSF/ITSR in Table 1 (Yang et al. 2010). Then the differentiation of two catfish species was analyzed and the primer pairs ITSPF/ITSPR were designed to amplify the specific regions (ITSP) of different sequences length based on the obtained ITS sequences.

**Table 1:** Information of primers pairs and sizes of the PCR products

Locus	Primer sequences (5′–3′)	PCR products sizes (bp)			
		*T. fulvidraco*	*F*_*TL*_	*L. Longirostris*	*F*_*LT*_
COI	F: CTACAATCCACCGCCTAA R: TAGAAGAAAGTGACAGAGCG	1515	1515	1515	1515
ITS	F: GTAGGTGAACCTGCGGAAGGATCA R: GAGTTTACCACCCGCTTTGGGCTGCATT	1106	–	1018	–
ITSP	F: CGTAACAAGGTTTCCGTAGGTG R: ATCCACCGCTAAGAGTTGTCAG	678	602 and 678	602	602 and 678

DNA amplifications were carried out by the polymerase chain reaction (PCR) in a total reaction volume of 25 μL. Each 25 μL PCR reaction system for *COI* and ITS contained 1 μL of 10 mM each primer, 1 U *Taq* DNA polymerase (TaKaRa, Japan), 2.5 μL 10 × PCR buffer (100 mM Tris–HCl, 500 mM KCl, 15 mM MgCl_2_; TaKaRa), 2 μL 10 mM dNTP (TaKaRa, Japan) and about 50 ng genomic DNA template. The PCR reaction for ITSP contained 1 μL of 10 mM each primer, 1.25 U *LA Taq* (TaKaRa, Japan), 12.5 μL 2 × GC bufferI (5 mM MgCl_2_, TaKaRa), 4 μL 10 mM dNTP (TaKaRa, Japan) and about 50 ng genomic DNA template. All PCR reactions were performed on S1000™ Thermal Cycler (BIO-RAD, USA) based on the different conditions. The procedures for *COI*: pre-denaturing at 94 °C for 5 min; 35 cycles of denaturing at 94 °C for 30 s, annealing at 52 °C for 30 s, and extending at 72 °C for 2 min; and a final extension at 72 °C for 10 min. The procedures for ITS: pre-denaturing at 94 °C for 5 min; 35 cycles of denaturing at 94 °C for 30 s, annealing at 54 °C for 30 s, and extending at 72 °C for 45 s; and a final extension at 72 °C for 10 min. The procedures for ITSP: pre-denaturing at 94 °C for 1 min; 30 cycles of denaturing at 94 °C for 30 s, annealing at 60 °C for 30 s, and extending at 72 °C for 2 min; and a final extension at 72 °C for 5 min.

The PCR products (*COI* and ITS) were run on 2.0% agarose gels for 1 h at 80 V and then purified by a DNA Agarose Gel Extraction Kit (Axygen, USA). The purified PCR products were cloned into pMD 18-T vector and sequenced by ABI 3730 automated sequencer (Applied Biosystems, USA).

### Data analysis

The obtained *COI* and ITS sequences were edited and aligned using the Clustal W (Hall 1999). A neighbor-joining (NJ) tree was constructed based on the *COI* gene sequences of all individuals from each group using MEGA Version 6 (Tamura et al. 2013). *Silurus meridionalis* was used as outgroup. The uncorrected p-distances model was carried out and node support was assessed based on 1000 bootstrap replicate. The different regions of ITS sequences in length were identified using MEGA Version 6 (Tamura et al. 2013). The primer pairs ITSPF/ITSPR (Table 1) were designed to amplify the specific ITS sites of *T. fulvidraco* and *L. longirostris*.

### DNA fragment size analysis

DNA fragment sizes of ITSP were determined by electrophoresis on 2% agarose gels with ethidium bromide (1 ng/mL) for 1 h at 80 V. The agarose gels were observed and captured by Gel document system (Clinx, China).

## Results

**Fig. 1:**
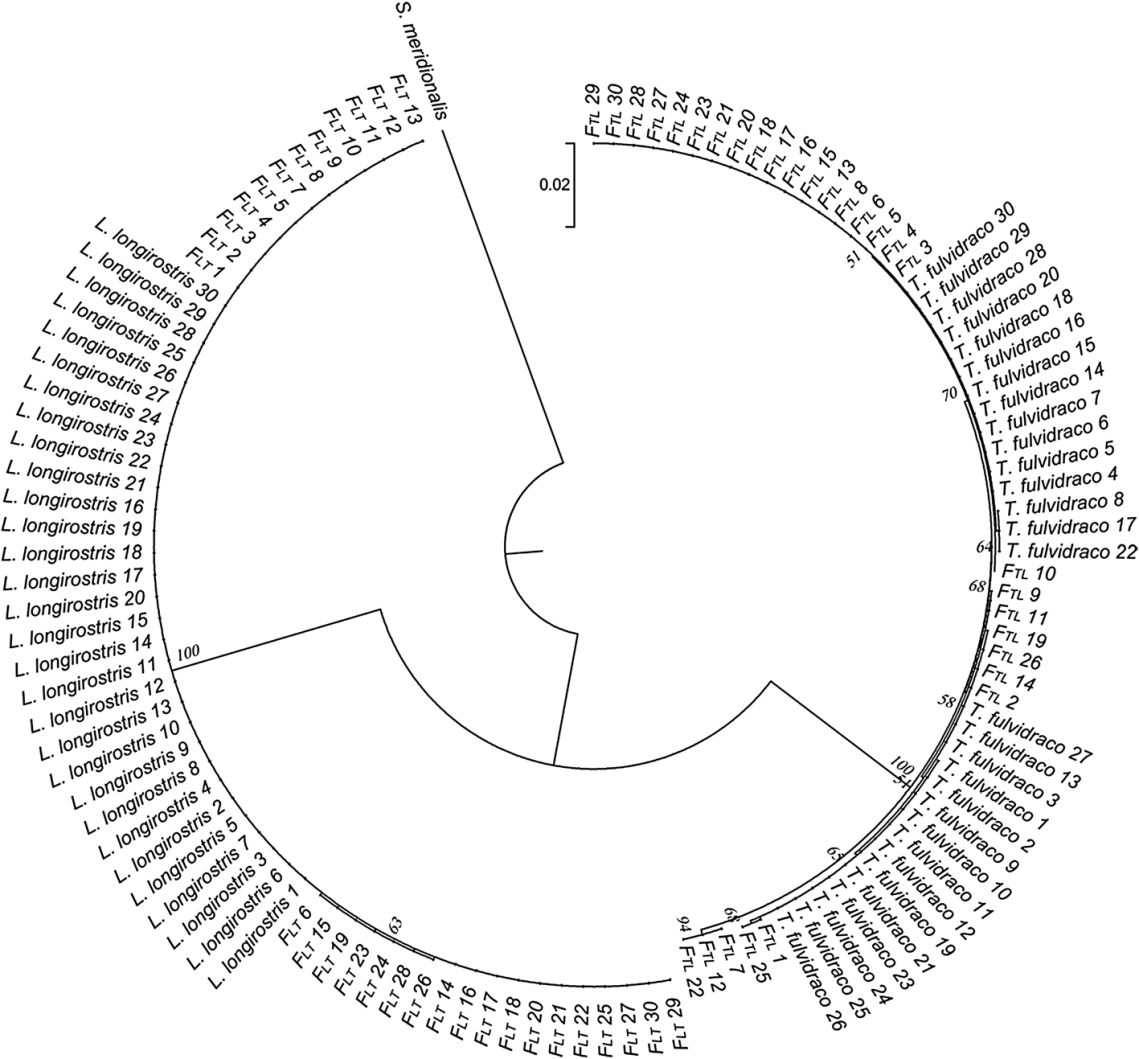
Phylogenetic tree based on *COI* sequences of *Tachysurus fulvidraco*, *Leiocassis longirostris* and their hybrids. The dendrogram was constructed based on the *COI* gene sequences by the neighbour-joining method. *Silurus meridionalis* was used as outgroup. F_TL_ *T. fulvidraco*(♀) × *L. longirostris*(♂) F_LT_ *L. longirostris*(♀) × *T. fulvidraco*(♂) 1–30 represents individual number of each population

To identify the maternal parentage, all individuals were amplified using the primers COIF/COIR for gene *COI*. For the *COI* sequences, the fragment sizes were approximately 1500 bp and there was no obvious difference in length among *T. fulvidraco*, *L. longirostris*, F_TL_ and F_LT_ (Table 1). Sequence analysis indicated that all individuals were divided into two groups, with one group including all the individuals of *T. fulvidraco* and hybrid F_TL_, and the other containing the rest *L. longirostris* and hybrid F_LT_. Similarly, the results showed that all individuals were divided into two clades as the former sequences analysis from phylogeny tree analyses (Fig. 1). The hybrid F_TL_ and *T. fulvidraco* were grouped into one clade, and the hybrid F_LT_ and *L. longirostris* into the other clade. These results suggested that all individuals came from two different maternal parentages, *T. fulvidraco* and *L. longirostris,* due to the maternal inheritance characteristics of mitochondrial. However, paternal line could not be identified from the sequences of *COI* gene.

**Fig. 2:**
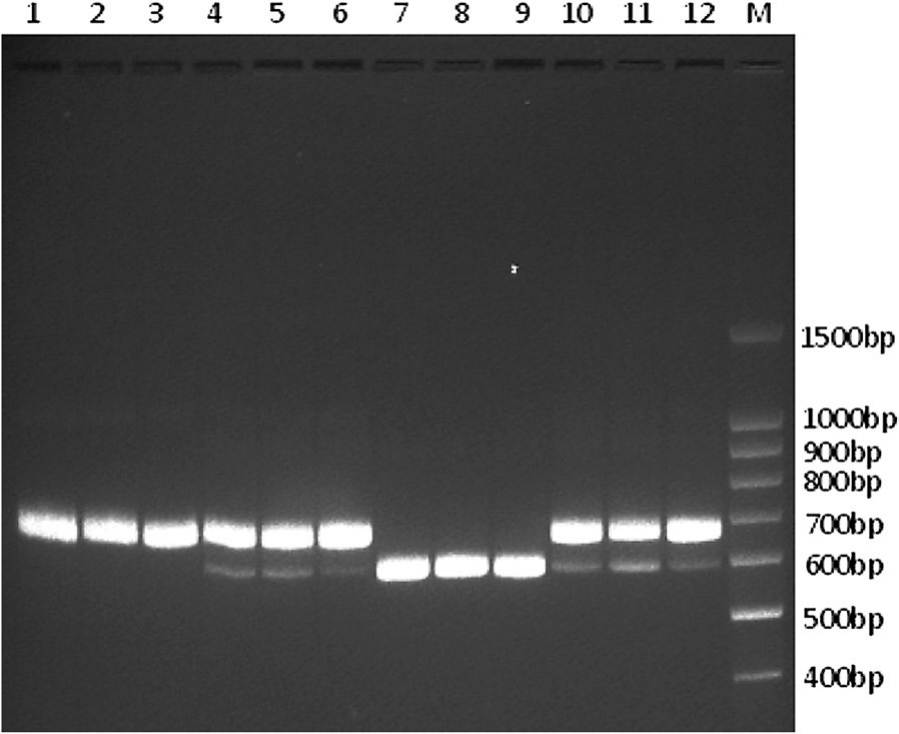
Electrophoresis patterns of the species-specific ITSP primers for ITS region of hybrids identification. *1–3* *T. fulvidraco* *4–6* F_TL_: *T. fulvidraco*(♀) × *L. longirostris* (♂) *7–9* *L. longirostris* *10–12* F_LT_: *L. longirostris*(♀) × *T. fulvidraco*(♂) M molecular marker. Two types of ITS sequence existed in hybrid individuals, one type of ITS is *T. fulvidraco*, and the other is *L. longirostris*

To detect the paternal line, ITSF/ITSR was firstly used to amplify the ITS sequences and to differentiate between *T. fulvidraco* and *L. longirostris*. By comparing the specific sites, some valuable regions were found. Then the primer pairs ITSPF/ITSPR were designed to obtain the amplification of the specific region for ITS (ITSP) based on their different sequences. Furthermore, all individuals were amplified and then detected by electrophoresis in 2% agarose gel. The results showed that there was only one band for *T. fulvidraco* individuals (about 670 bp) and *L. longirostris* individuals (about 600 bp) (Fig. 2), while a heterozygous pattern with two bands (600 and 670 bp) was observed for hybrids F_TL_ and hybrids F_LT_, respectively. Together with the result of maternal line, the paternal line could thus be inferred (Fig. 2).

In conclusion, a method based on sequence variation was established for taxonomic identification of hybrids. Firstly, we used the mitochondrial *COI* primers to obtain the sequences of the individuals and construct phylogeny tree to determine their maternal parentage by analyzing their homology and phylogenic relation. Then we used the nuclear marker ITSPF/ITSPR to detect the hybrids and their paternal line. The application of this mitochondrial *COI* and internal transcribed spacer ITS marker has been proved to be feasible to identify the hybrids.

## Discussion

Hebert et al. (2003) proposed a molecular technique named DNA barcoding to identify species and *COI* gene has then been commonly used as a standardized marker. Though *COI* gene is widely used as a genetic marker for fish species authentication, it is not an appropriate marker for all aspects of species identification (Clark 2015). The *COI* gene sequences only could not determine a hybrid because mtDNA exhibits maternal inheritance. The *COI* gene of hybrid F1 showed identical characteristic with its maternal parentage and can not represent different alleles (Rubinoff et al. 2006). Thus, hybrid individuals could be misidentified as their maternal parent species based solely on *COI* gene. Other genetic markers must be further considered due to the potential problem for accurate identification based on single *COI* gene (Rubinoff et al. 2006).

Recently, some researches have been carried out to identify hybrids and detect the hybridization events based on the mitochondrial and nuclear molecular markers. Hashimoto et al. (2011) established the molecular appraised technology for Serrasalmid fish and their hybrids based on mitochondrial genes (*COI* and *Cytb*) and nuclear genes (*RAG2*). The molecular diagnostic method was also established for hybrids between Netropical catfish species *Pseudoplatystoma corruscans* and *Pseudoplatystoma reticulatum* by mitochondrial 16S and nuclear genes *RAG2* (Prado et al. 2011). Dio et al. (2015) identified the hybridization of burrfish between *Chilomycterus antillarum* and *Chilomycterus schoepfii* using *COI* gene sequences and AFLP technology. The interspecies hybridization in the freshwater stingrays *Potamotrygon motoro* and *P. falkneri* was revealed by mitochondrial gene (*COI* and *Cytb*) and nuclear microsatellite markers (Cruz et al. 2015). The results show that the established identification methods can be rapidly implemented and effectively determine the hybrid individuals. The ITS region exhibited low variations within species and high variations between species (Yu et al. 2006). What is more, it has several advantages due to its rapid evolution, easy isolation and non-coding structure (Chow et al. 2009). It has been widely used in the molecular identification and systematics studies for species discrimination (Chow et al. 2009; Yang et al. 2010; Zhu et al. 2011). As a result of hybridization, the nuclear ITS locus is a heterozygote consisted of heterologous alleles, one of which comes from maternal line and the other from paternal line for diploid individuals. Hence, it could be used to successfully identify hybrids by the heterozygous pattern in this study.

Primer design must be considered as a robust diagnostic technology. First, the conservative and consistent sites were used to design the primer pairs to simultaneously achieve the specific ITSP region sequences of *T. fulvidraco* individuals, *L. longirostris* and their hybrids. Second, there were different length PCR products by amplification of designed species-specific primers, and the species-diagnostic bands could be easily distinguished (Pank et al. 2001). In this study, the amplification sequences gained for *T. fulvidraco* individuals and *L. longirostris* individuals by primers ITSPF/ITSPR were approximately 670 and 600 bp, respectively, which could be easily and directly observed from the agarose gel. Another consideration factor was to ensure the high efficiency of the amplification reaction (Pank et al. 2001). In our study, we increased the amplification success rates and simultaneously obtained two different-sized products by utilization of *LA Taq* and GC buffer.

Establishing identification technology for hybrids is vital to monitor hybridization programs and manage crossbreeding progress (Alllendorf et al. 2001). Meanwhile, hybridization programs should be continuously monitored to assess their impacts and safeguard wild populations since it is almost impossible to recover the population characteristics if the wild populations were suffered from genetic contamination. In this study, molecular diagnostic method for the target fish species was successfully established, and it could be applied for identifying not only eggs, larvae and young individuals during the breeding procedure but also fish meat and fish products. It could also become a reference for establishing detection technique for hybrids in other groups of fishes.

## Conclusions

The molecular identification methods for *T. fulvidraco*, *L. Longirostris* and their hybrids were successfully established. Meanwhile, these molecular diagnostic tools could also be used routinely to assess breeding plans of fish farms, better manage fish hybrids, as well as to monitor and minimize the negative impacts resulting from the implementation of hybridization projects in aquaculture industry.
